# Biomarkers of systemic inflammation predict survival with first-line immune checkpoint inhibitors in non-small-cell lung cancer

**DOI:** 10.1016/j.esmoop.2022.100445

**Published:** 2022-04-07

**Authors:** M. Stares, T.E. Ding, C. Stratton, F. Thomson, M. Baxter, H. Cagney, K. Cumming, A. Swan, F. Ross, C. Barrie, K. Maclennan, S. Campbell, T. Evans, A. Tufail, S. Harrow, H. Lord, B. Laird, M. MacKean, I. Phillips

**Affiliations:** 1 Edinburgh Cancer Centre, NHS Lothian, Western General Hospital, Edinburgh; 2 University of Edinburgh, Cancer Research UK Edinburgh Centre, Institute of Genetics and Cancer, Western General Hospital, Edinburgh; 3 Division of Molecular and Clinical Medicine, Ninewells Hospital and Medical School, University of Dundee, Dundee; 4 Tayside Cancer Centre, Ninewells Hospital and Medical School, NHS Tayside, Dundee; 5 School of Medicine, Ninewells Hospital and Medical School, University of Dundee, Dundee, UK

**Keywords:** non-small-cell lung cancer (NSCLC), immune checkpoint inhibitors, inflammation, biomarker, prognosis, Scottish Inflammatory Prognostic Score (SIPS)

## Abstract

**Introduction:**

Pembrolizumab is an established first-line option for patients with advanced non-small-cell lung cancer (NSCLC) expressing programmed death-ligand 1 ≥50%. Durable responses are seen in a subset of patients; however, many derive little clinical benefit. Biomarkers of the systemic inflammatory response predict survival in NSCLC. We evaluated their prognostic significance in patients receiving first-line pembrolizumab for advanced NSCLC.

**Methods:**

Patients treated with first-line pembrolizumab for advanced NSCLC with programmed death-ligand 1 expression ≥50% at two regional Scottish cancer centres were identified. Pretreatment inflammatory biomarkers (white cell count, neutrophil count, neutrophil/lymphocyte ratio, platelet/lymphocyte ratio, albumin, prognostic nutritional index) were recorded. The relationship between these and progression-free survival (PFS) and overall survival (OS) were examined.

**Results:**

Data were available for 219 patients. On multivariate analysis, albumin and neutrophil count were independently associated with PFS (*P* < 0.001, *P* = 0.002, respectively) and OS (both *P* < 0.001). A simple score combining these biomarkers was explored. The Scottish Inflammatory Prognostic Score (SIPS) assigned 1 point each for albumin <35 g/l and neutrophil count >7.5 × 10^9^/l to give a three-tier categorical score. SIPS predicted PFS [hazard ratio 2.06, 95% confidence interval (CI) 1.68-2.52 (*P* < 0.001)] and OS [hazard ratio 2.33, 95% CI 1.86-2.92 (*P* < 0.001)]. It stratified PFS from 2.5 (SIPS2), to 8.7 (SIPS1) to 17.9 months (SIPS0) (*P* < 0.001) and OS from 5.1 (SIPS2), to 12.4 (SIPS1) to 28.7 months (SIPS0) (*P* < 0.001). The relative risk of death before 6 months was 2.96 (95% CI 1.98-4.42) in patients with SIPS2 compared with those with SIPS0-1 (*P* < 0.001).

**Conclusions:**

SIPS, a simple score combining albumin and neutrophil count, predicts survival in patients with NSCLC receiving first-line pembrolizumab. Unlike many proposed prognostic scores, SIPS uses only routinely collected pretreatment test results and provides a categorical score. It stratifies survival across clinically meaningful time periods that may assist clinicians and patients with treatment decisions. We advocate validation of the prognostic utility of SIPS in this and other immune checkpoint inhibitor treatment settings.

## Highlights

• Biomarkers of systemic inflammation are strong prognostic factors in patients with NSCLC.
• The Scottish Inflammatory Prognostic Score is a simple score combining albumin and neutrophil count.
• SIPS objectively stratifies survival of patients in a clinically meaningful timeframe.

## Introduction

Lung cancer is the leading cause of cancer death around the world, claiming 1.8 million lives in 2020.^1^ This is despite significant advances in the treatment of this disease over the last decade. In particular, immune checkpoint inhibitors (ICIs) have emerged for the treatment of non-small-cell lung cancers (NSCLC) which represent 85% of all cases. Since the first US Food and Drug Administration license in 2015 the anti-programmed cell death protein 1 (anti-PD-1) agents (i.e. pembrolizumab,^2^, ^3^, ^4^, ^5^ nivolumab^6^, ^7^, ^8^) and anti-programmed death-ligand 1 (anti-PD-L1) agents (atezolizumab,^9^^,^^10^ durvalumab^11^) have rapidly become ‘standard of care’ treatment in multiple indications.

Pembrolizumab monotherapy is a standard of care first-line treatment option for patients with advanced NSCLC expressing PD-L1 ≥50%. The KEYNOTE-024 study demonstrated improved progression-free survival (PFS) and overall survival (OS) with pembrolizumab monotherapy compared with platinum-based chemotherapy in this setting.^3^ Pembrolizumab monotherapy led to a doubling of the 5-year OS rate relative to chemotherapy (31.9% versus 16.3%, respectively). Not all eligible patients responded to first-line pembrolizumab monotherapy, however, with objective response rates only 44.8%, and approximately 20% of patients died within 6 months of starting treatment.

In addition to PD-L1 status, a number of genomic and immune predictive biomarkers of response to ICI therapy in NSCLC, such as: tumour mutational burden, presence of genomic alterations in DNA damage response and other specific gene pathways, neoantigenic load, immune gene expression signatures, and immune features of the tumour microenvironment, have been investigated.^12^^,^^13^ To date, these remain largely experimental and not readily available in a time- or cost-efficient manner for routine clinical practice. Even before this era of ICIs, pretreatment biomarkers of the systemic inflammatory response and malnutrition have been recognised as independent prognostic biomarkers in many cancer types, including NSCLC.^14^^,^^15^ Baseline values of peripheral blood components [e.g. white cell count (WCC), neutrophil count (NC), lymphocyte count (LC), platelet count, serum albumin, C-reactive protein (CRP), lactate dehydrogenase (LDH)], either individually or as composite prognostic scores [e.g. neutrophil/lymphocyte ratio (NLR), platelet/lymphocyte ratio (PLR), prognostic nutritional index (PNI), lung immune prognostic index (LIPI), the modified Glasgow Prognostic Score (mGPS) etc] have been investigated as prognostic biomarkers in patients with NSCLC receiving ICIs.^14^, ^15^, ^16^, ^17^, ^18^, ^19^, ^20^, ^21^, ^22^ Using readily available, cheap, standard investigations, these simple biomarkers may be used alongside clinical assessments to provide additional objective information when making treatment decisions. None, however, are yet routinely used in standard clinical practice. Reasons for this include a lack of independent validation, assessment outside clinical trial populations, and direct comparison between biomarkers. Studies have also applied different normal/abnormal thresholds, further hampering comparisons.

The Edinburgh and National Health Service Tayside Cancer Centres provide regional cancer services in Scotland, UK, serving a population of ∼2 million. We sought to utilise real-world experience to identify and compare the prognostic value of peripheral blood biomarkers collected as part of the routine clinical care of patients with advanced NSCLC receiving first-line ICI monotherapy.

## Materials and methods

### Study population

All patients being treated with first-line pembrolizumab monotherapy for advanced NSCLC by the Edinburgh or Tayside Cancer Centres between June 2016 and January 2021 were identified from the electronic prescribing record. Eligible patients were 18 years or over, had a pathological diagnosis of NSCLC, a PD-L1 status ≥50% and had received at least one dose of pembrolizumab therapy.

### Procedure and assessment

Patient demographics and pathological data were recorded. Eastern Cooperative Oncology Group performance status (ECOG PS), WCC, NC, LC, platelets, and albumin within 14 days before, and as near to, cycle 1, day 1 (C1D1) pembrolizumab monotherapy were recorded. NLR [NC (×10^9^/l)/LC (×10^9^/l)], PLR [platelets (×10^9^/l)/LC (×10^9^/l)], and PNI [albumin (g/l) + 5 × LC (×10^9^/l)] were calculated. WCC, NC, and albumin were categorised within normal limits, in line with previous work in this area.^15^^,^^23^^,^^24^ Cut-offs for NLR (≤/>5),^17^^,^^22^ PLR (≤/>180),^25^^,^^26^ and PNI (≥/<45)^21^^,^^27^ were based on previous studies examining these factors and not derived from the present analysis.

All data were collected as part of routine oncology work up in keeping with standard of care. No patient identifiable data were used. As the study was not designed to test a formal hypothesis, a sample size calculation was not required; all patients treated during the aforementioned time period were assessed.

### Statistical analysis

PFS, defined as the number of months from C1D1 pembrolizumab until radiological or clinical evidence of progressive disease prompting cessation of treatment, or censorship (10/05/2021) if no evidence of progressive disease at follow-up date, was calculated. OS, defined as the number of months from C1D1 pembrolizumab until death, or censorship (10 May 2021) if alive at follow-up date, was calculated.

Survival curves were plotted using Kaplan–Meier methods, and the log-rank test applied. Survival analysis was carried out using Cox’s proportional-hazards model, and hazard ratios (HRs) were calculated. Multivariate survival analysis was carried out using a stepwise backward procedure to derive a final model of the variables that had a significant independent relationship with survival. To remove a variable from the model, the corresponding *P* value had to be >0.10.

All analyses were carried out in SPSS Version 24.0 (SPSS Inc., Portsmouth, Hampshire, UK). The study adheres to the Reporting Recommendations for Tumour Marker Prognostic Studies guideline.

## Results

A total of 219 patients were identified (Table 1). Demographic, biomarker, and survival data were available for all patients. The median age was 69 (61-73) years and 50% were female. The majority [*n* = 172 (79%)] of patients had non-squamous histologic subtype. Median PFS was 7.6 [interquartile range (IQR) 2.1-21.0] months. At the time of censoring 57 (26%) of patients had no evidence of progressive disease. The minimum and median follow-up of these patients was 5.4 months and 20.0 months, respectively. Median OS was 12.1 (IQR 4.3-31.0) months. At the time of censoring, 79 (37%) patients were alive. The minimum and median follow-up of survivors was 4.5 and 19.8 months, respectively.

**Table 1:** Clinical characteristics and survival in patients with programmed death-ligand 1+ non-small-cell lung cancer receiving first-line pembrolizumab monotherapy: univariate log-rank analysis

		*n* (%)	Progression-free survival		Overall survival	
			Median (IQR)	*P*	Median (IQR)	*P*
**All**		219	7.6 (2.1-21.0)	**n/a**	12.1 (4.3-31.0)	**n/a**
Age, years	≤64	84 (38)	3.6 (1.3-15.6)	***0.001***	9.7 (2.2-19.4)	***0.004***
	65-74	107 (49)	9.4 (3.2-35.1)		15.8 (6.6-n/r)	
	>74	28 (13)	11.8 (2.5-17.5)		12.1 (4.3-21.3)	
Sex	Female	110 (50)	8.1 (2.0-20.2)	*0.906*	11.0 (4.8-28.7)	*0.669*
	Male	109 (50)	7.1 (2.2-21.8)		12.9 (4.3-31.0)	
ECOG performance status	0	29 (13)	11.9 (3.7-n/r)	***0.001***	28.7 (7.0-n/r)	***<0.001***
	1	147 (67)	8.2 (2.5-22.1)		15.2 (5.7-n/r)	
	2	43 (20)	3.2 (1.3-10.0)		7.3 (2.1-12.1)	
Histologic subtype	Squamous Carcinoma	47 (21)	9.8 (2.9-16.4)	*0.766*	12.4 (3.5-31.0)	*0.848*
	Non-squamous	172 (79)	7.1 (1.8-22.1)		11.0 (7.4-23.9)	
White cell count	≤11.0 × 10^9^/l	132 (60)	12.1 (2.8-33.5)	***<0.001***	16.8 (7.8-n/r)	***<0.001***
	>11.0 × 10^9^/l	87 (40)	3.6 (1.6-10.4)		7.4 (2.2-17.6)	
Neutrophil count	≤7.5 × 10^9^/l	119 (54)	15.0 (4.4-n/r)	***<0.001***	21.3 (9.8-n/r)	***<0.001***
	>7.5 × 10^9^/l	100 (46)	3.2 (1.5-9.0)		6.8 (2.2-15.2)	
NLR	<5	111 (51)	13.1 (4.0-33.5)	***<0.001***	20.5 (8.8-n/r)	***<0.001***
	≥5	108 (49)	3.5 (1.3-13.2)		7.6 (2.2-16.8)	
PLR	≤180	58 (27)	12.1 (3.7-22.1)	***0.049***	17.9 (9.7-31.0)	***0.030***
	>180	161 (74)	6.0 (1.9-19.7)		9.9 (3.3-28.7)	
Albumin	≥35 g/l	96 (44)	15.0 (5.7-n/r)	***<0.001***	28.7 (10.7-n/r)	***<0.001***
	<35 g/l	123 (56)	3.6 (1.6-13.2)		7.7 (2.5-16.8)	
**PNI**	**≥45**	68 (31)	15.0 (3.8-35.1)	***0.001***	28.7 (12.1-n/r)	***<0.001***
	**<45**	151 (69)	5.1 (1.8-17.2)		9.0 (2.8-21.4)	

Bold and italic values are statistical significance.

ECOG, Eastern Cooperative Oncology Group; IQR, interquartile range; n/a, not appropriate; n/r, not reached; NLR, neutrophil/lymphocyte ratio; PLR, platelet/lymphocyte ratio; PNI, prognostic nutritional index.

The relationship between prognostic factors and PFS was examined (Table 2). On univariate analysis, age (*P* = 0.011), PS (*P* < 0.001), WCC (*P* < 0.001), NC (*P* < 0.001), NLR (*P* < 0.001), albumin (*P* < 0.001), and PNI (*P* = 0.001) were predictive of PFS. On multivariate analysis, only albumin (*P* < 0.001), NC (*P* = 0.002), and PS (*P* = 0.019) were independently predictive of PFS. Albumin stratified PFS from 3.6 (IQR 1.6-13.2) months (albumin <35 g/l) to 15.0 (IQR 5.7-50.4) months (albumin ≥35 g/l) (*P* < 0.001). NC stratified PFS from 3.2 (IQR 1.5-9.0) months (NC >7.5 × 10^9^/l) to 15.0 (IQR 4.4-not reached) months (NC ≤7.5 × 10^9^/l) (*P* < 0.001).

**Table 2:** The relationship between prognostic factors and progression-free survival or overall survival in patients with non-small-cell lung cancer receiving first-line pembrolizumab monotherapy: univariate and multivariate Cox-regression analysis

	Progression-free survival				Overall survival			
	Univariate		Multivariate		Univariate		Multivariate	
	HR (95% CI)	*P*	HR (95% CI)	*P*	HR (95% CI)	*P*	HR (95% CI)	*P*
Age, years (≤64, 65-74, ≥75)	**0.73 (0.57-0.93)**	***0.011***			0.82 (0.63-1.06)	*0.815*		
Sex (male, female)	1.02 (0.75-1.39)	*0.901*			1.08 (0.77-1.50)	*0.670*		
Performance status (0, 1, 2)	**1.71 (1.28-2.27)**	***<0.001***	**1.48 (1.11-1.98)**	***0.019***	**1.87 (1.37-2.55)**	***<0.001***	***1.48 (1.06-2.06)***	***0.021***
Histological subtype (squamous, non-squamous)	1.06 (0.73-1.54)	*0.767*			1.17 (0.91-1.50)	*0.229*		
WCC (≤11 × 10^9^/l, >11 × 10^9^/l)	**1.92 (1.40-2.63)**	***<0.001***			**2.21 (1.58-3.09)**	***<0.001***		
Neutrophils (≤7.5 × 10^9^/l, >7.5 × 10^9^/l)	**2.47 (1.80-3.38)**	***<0.001***	**1.73 (1.22-2.44)**	***0.002***	**2.92 (2.07-4.11)**	***<0.001***	**2.10 (1.48-3.03)**	***<0.001***
Neutrophil/lymphocyte ratio (≤5, >5)	**2.03 (1.49-2.78)**	***<0.001***			**2.39 (1.70-3.36)**	***<0.001***		
Platelet/neutrophil ratio (≤180, >180)	1.44 (1.00-2.07)	*0.052*			**1.55 (1.04-2.32)**	***0.031***		
Albumin (<35 g/l, ≥35 g/l)	**2.56 (1.84-3.58)**	***<0.001***	**2.11 (1.48-3.00)**	***<0.001***	**3.06 (2.11-4.44)**	***<0.001***	**2.29 (1.55-3.39)**	***<0.001***
Prognostic nutritional index (<45, ≥45)	**1.87 (1.30-2.69)**	***0.001***			**2.70 (1.75-4.17)**	***<0.001***		

Bold and italic values are statistically significant.

CI, confidence interval; HR, hazard ratio; WCC, white cell count.

On univariate analysis, PS (*P* < 0.001), WCC (*P* < 0.001), NC (*P* < 0.001), NLR (*P* < 0.001), platelets (*P* = 0.011), PLR (*P* = 0.031), albumin (*P* < 0.001), and PNI (*P* < 0.001) were predictive of OS. On multivariate analysis, only albumin (*P* < 0.001), NC (*P* < 0.001), and PS (*P* = 0.021) were independently predictive of OS. Albumin stratified OS from 7.7 (IQR 2.5-16.8) months (albumin <35 g/l) to 28.7 (IQR 10.7-not reached) months (albumin ≥35 g/l) (*P* < 0.001). NC stratified OS from 6.8 (IQR 2.20-15.2) months (NC >7.5 × 10^9^/l) to 21.3 (IQR 9.8-not reached) months (NC ≤7.5 × 10^9^/l) (*P* < 0.001).

Given the consistent, highly significant relationship between the objective biomarkers of systemic inflammation, albumin, and NC with both PFS and OS, a simple cumulative score combining these factors was explored. The Scottish Inflammatory Prognostic Score (SIPS) assigned a point to each of albumin <35 g/l and NC >7.5 × 10^9^/l to give a three-tier score: 0 = low risk, 1 = moderate risk, 2 = high risk (Table 3).

**Table 3:** Description of the Scottish Inflammatory Prognostic Score

Description		Scottish Inflammatory Prognostic Score
Albumin	Neutrophil count	
≥35 g/l	≤7.5 × 10^9^/l	0
≥35 g/l	>7.5 × 10^9^/l	1
<35 g/l	≤7.5 × 10^9^/l	1
<35 g/l	>7.5 × 10^9^/l	2

**Figure 1:**
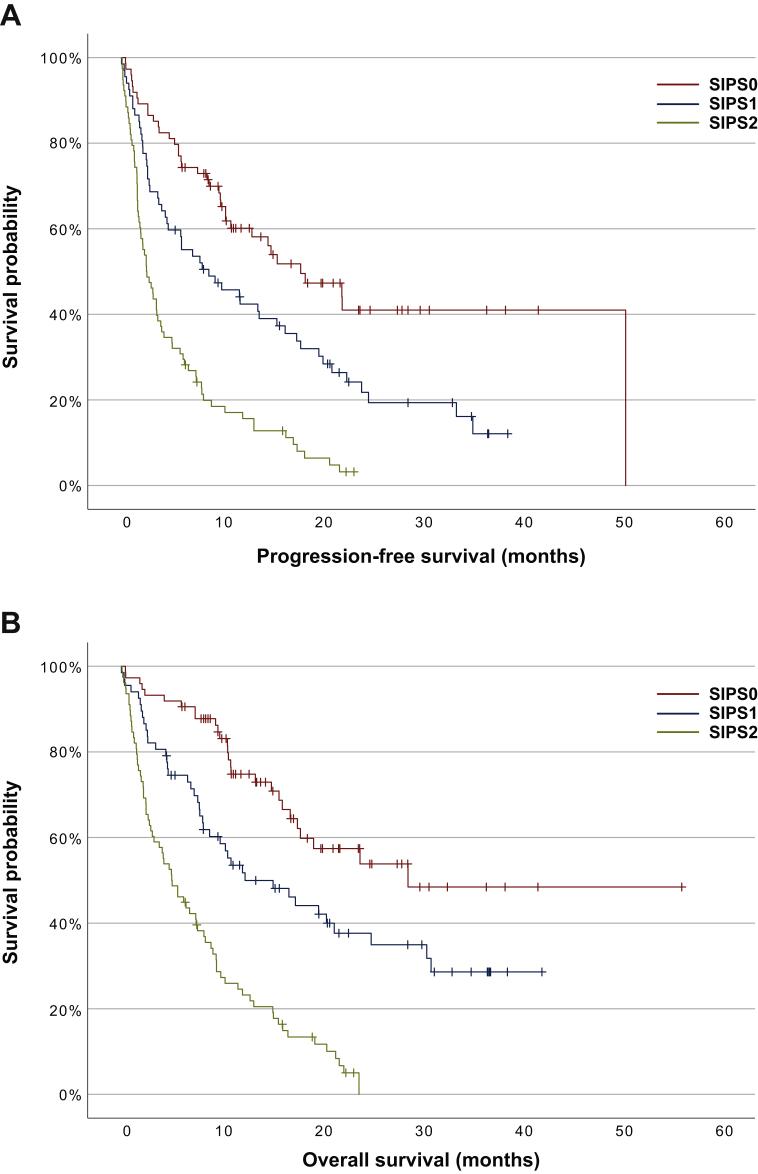
Kaplan–Meier survival curves examing the relationship between the Scottish Immunotherapy Prognostic Score (SIPS) and (A) progression-free survival and (B) overall survival in patients with non-small-cell lung cancer receiving first-line pembrolizumab monotherapy.

The distribution of SIPS across the cohort was broadly even: SIPS0 *n* = 74 (34%), SIPS1 *n* = 67 (31%), SIPS2 *n* = 78 (36%) (Table 4). Patients with SIPS2 were more frequently PS2+ than those with SIPS0 or 1 [29% versus 14% (*P* = 0.003)]. SIPS was predictive of both PFS [HR 2.06, 95% CI 1.68-2.52 (*P* < 0.001)] and OS [HR 2.33, 95% CI 1.86-2.92 (*P* < 0.001)]. SIPS stratified PFS from 2.5 (IQR 1.3-7.5) months (SIPS2), to 8.7 (IQR 2.6-22.5) months (SIPS1), to 17.9 (IQR 4.0-50.4) (SIPS0) months (*P* < 0.001) and OS from 5.1 (IQR 1.9-11.7) months (SIPS2), to 12.4 (IQR 4.7-not reached) months (SIPS 1), to 28.7 (IQR 11.0-not reached) months (SIPS0) (*P* < 0.001) (Figure 1). The 1-year survival was 47% and 43% in patients with SIPS0 and SIPS1, respectively, but only 12% in those with SIPS2 (*P* = 0.001). The relative risk of death before 6 months was 2.96 (95% CI 1.98-4.42) (*P* < 0.001) in patients with SIPS2 compared with those with SIPS0 or 1.

**Table 4:** The relationship between Scottish Inflammatory Prognostic Score (SIPS) and progression-free survival and overall survival at 3 months, 6 months, and 12 months in patients with non-small-cell lung cancer receiving first-line pembrolizumab monotherapy: *P* < 0.001 log rank

	SIPS	Patients [*n* (%)]	Median (IQR) months	*P*	Survival at 3 months [*n* (%)]	Survival at 6 months [*n* (%)]	Survival at 12 months [*n* (%)]
**PFS**	0	74 (34)	17.9 (6.0-50.4)	*<0.001*	64 (86)	56 (76)	31 (42)
	1	67 (31)	8.7 (2.6-22.5)		46 (68)	38 (57)	25 (37)
	2	78 (36)	2.5 (1.3-7.5)		36 (46)	24 (31)	12 (15)
**OS**	0	74 (34)	28.7 (11.0-n/r)	*<0.001*	69 (93)	68 (92)	41 (55)
	1	67 (31)	12.4 (4.7-n/r)		55 (82)	47 (70)	30 (45)
	2	78 (36)	5.1 (1.9-11.7)		48 (62)	36 (46)	18 (23)

Italic values are statistically significant.

IQR, interquartile range; n/r = not reached; OS, overall survival; PFS, progression-free survival.

SIPS was predictive of both PFS [HR 1.98, 95% CI 1.29-3.04 (*P* = 0.002)] and OS [HR 2.47, 95% CI 1.49-4.11 (*P* < 0.001)] in the squamous NSCLC cohort. In the non-squamous NSCLC cohort, SIPS was also predictive of both PFS [HR 2.05, 95% CI 1.62-2.58 (*P* < 0.001)] and OS [HR 2.30, 95% CI 1.79-3.00 (*P* < 0.001)]. KRAS mutation status was available for 130/172 cases of non-squamous NSCLC. In these patients, KRAS mutation status was not predictive of PFS [HR 0.90, 95% CI 0.59-1.36 (*P* = 0.897)] or OS [HR 0.98, 95% CI 0.62-1.53 (*P* = 0.643)].

## Discussion

This study has demonstrated that biomarkers of systemic inflammation and malnutrition independently predict survival in patients with NSCLC expressing PD-L1 ≥50% treated with first-line pembrolizumab monotherapy. In particular, pretreatment serum albumin and NC, either individually or as part of the novel SIPS, may provide useful prognostic information to help clinicians and patients when making treatment decisions and guide ‘realistic medicine’ conversations.

The relationship between inflammation and cancer has been under investigation since Virchow hypothesized that cancers arose at the sites of chronic inflammation.^28^ We now recognise that inflammation and the immune system play key roles in the development, survival, and progression of cancer.^29^ Further, our understanding of the ability of cancers to evade immune destruction gave rise to the development and use of ICIs such as pembrolizumab.^3^^,^^30^^,^^31^ Although many of these interactions takes place within the tumour microenvironment, systemic inflammatory effects are also observed. Circulating blood biomarkers of systemic inflammation are routinely measured in clinical practice and their prognostic significance in cancer is now well established. This has given rise to numerous prognostic scores in cancer. We provide further validatory evidence for the prognostic value of NLR, PLR, and PNI in patients with NSCLC.

To date, NLR is perhaps the best studied biomarker of systemic inflammation in NSCLC. Neutrophils play an important role in tumour growth and progression via direct effects on cancer cells or indirectly via influences on the tumour microenvironment.^32^, ^33^, ^34^ Raised NC has been linked to poorer NSCLC survival.^18^^,^^35^^,^^36^ As part of the NLR, an increase in the NC and/or decrease in the LC (i.e. high NLR) indicates a reduced antitumour effect of the immune system and poor response to ICI.^16^^,^^17^^,^^22^^,^^35^^,^^37^ In our cohort, NLR was predictive of survival on univariate analysis only, stratifying OS from 7.6 (IQR 2.2-16.8) months (NLR ≥5) to 20.5 (IQR 8.8-not reached) months (NLR <5) (*P* < 0.001). A limiting factor of NLR, and other prognostic scores such as PLR, PNI, and LIPI, is significant variability between studies in the cut-off value applied.^16^^,^^17^^,^^26^^,^^37^^,^^38^ NLR ≥5 was applied in the current study as the most frequently used cut-off in these previous studies of NSCLC.^37^^,^^38^ NLR cut-offs in other studies, however, range between 2.11 and 6.5.^37^ It is also noted that NLR ≥3 is most commonly applied in studies of renal cell carcinoma outcome.^39^^,^^40^ These inter- and intra-tumour type variances significantly limit the ease and application of such scores in routine clinical practice.

NLR has also been combined with other biomarkers such as mGPS and platelets.^41^, ^42^, ^43^ The lung cancer-specific LIPI score, a combination of derived NLR and LDH, a generalised marker of tissue damage/inflammation, predicts survival of patients with NSCLC treated with ICIs.^19^^,^^20^ It is a limitation of this study that we were unable to assess LIPI or mGPS in our cohort, as baseline LDH and CRP are not routinely collected at our institutes. The mGPS, like PNI, utilises albumin as a marker of systemic inflammation. Inflammation and malnutrition both decrease the rate of albumin synthesis, whereas inflammation is also associated with increased albumin catabolism and influences albumin distribution between the vascular and extravascular components.^28^^,^^44^^,^^45^ Serum albumin concentrations are associated with survival in a range of medical conditions, including cancer.^14^^,^^15^^,^^45^, ^46^, ^47^ As part of the mGPS or PNI it holds prognostic value in patients with NSCLC or other cancers treated with immunotherapy.^14^^,^^15^^,^^21^^,^^27^^,^^48^^,^^49^ In our cohort, PNI stratified OS from 9.0 (IQR 2.8-21.4) months (PNI <45) to 28.7 (IQR 10.7-not reached) months (*P* < 0.001).

Given the importance of NC and serum albumin concentrations to these prognostic scores, it was unsurprising to find that they hold individual independent prognostic significance in this cohort. We propose a novel score which, to our knowledge, is the first to combine NC and albumin as key biomarkers of systemic inflammation. Unlike many other prognostic scores, SIPS uses only routinely collected pretreatment test results and normal reference values as cut-offs for its constituent factors. As a result, it is rapidly available, cheap, and easy to interpret. This will promote its incorporation into routine clinical practice. The use of normal reference value cut-offs for albumin and neutrophils is supported by previous work in this area.^15^^,^^23^^,^^24^ In particular, the mGPS, which utilises normal reference value cut-offs for albumin (≥35 g/l) and CRP (≤10 U/l), has been extensively studied and is associated with survival, quality of life, weight loss, and response to treatment in patients with cancer.^14^^,^^15^^,^^50^^,^^51^

Like the mGPS, SIPS provides a simple three-tier categorical score that stratifies survival across a clinically meaningful time period, providing more granular information than other binary scores. In particular, it more accurately identifies a group with SIPS2, representing approximately one-third of patients, for whom median PFS [2.5 (IQR 1.3-7.5) months] is particularly guarded. This time period is more clinically relevant than that identified in the binary poor prognosis groups for NC or albumin alone, being shorter than the 12 weeks at which a first radiological tumour assessment is typically carried out. Further, approximately one-third of patients with SIPS2 died within 3 months of starting treatment, with fewer than half alive at 6 months compared with 92% of those with SIPS0. These patients may benefit from earlier radiological or clinical assessment of treatment benefit to allow earlier identification of treatment futility. Alternatively, with this information these patients may opt not to pursue ICI monotherapy treatment. These outcomes would reduce the risk of treatment-related adverse events and facilitate more prompt referral to specialist palliative care services and discussions about appropriate end-of-life care. Additionally, alternative treatment options, such as combination cytotoxic chemotherapy and immunotherapy regimens, could be explored in patients with SIPS1 or SIPS2.^4^^,^^5^ Outcomes, however, were poor for patients with NSLC and high levels of systemic inflammation treated in the era of standard of care first-line cytotoxic chemotherapy, and emerging evidence suggests this may also be the case for combination chemoimmunotherapy.^14^^,^^15^^,^^48^^,^^52^^,^^53^

Conversely, a key tenet of ICI use in cancer is the opportunity for durable benefit. Although this term lacks formal definition, we note that to date only patients with SIPS0/1 have demonstrated PFS or OS >2 years. Indeed, 11/23 (48%) of patients with SIPS0 and 13/29 (45%) with SIPS1 who started treatment >2 years before the censor date, are still alive. Both PFS and OS were shorter in this cohort than that reported in clinical trial cohorts, including the pivotal KEYNOTE-024 study.^3^^,^^54^ Survival in our cohort is, however, similar to that reported in other real-world cohorts.^55^, ^56^, ^57^ This likely reflects the inclusion of patients who would not have been eligible for the KEYNOTE-024 study, including those with poor ECOG PS (i.e. ≥2) or brain metastases. PS is the most widely validated prognostic indicator in cancer and it again showed independent prognostic significance in this cohort. It is a highly subjective measure, however, prone to bias and associated with interindividual variation and overestimation compared with patient-reported assessments.^58^^,^^59^ It is also rarely available at the time of multidisciplinary team meetings.^59^ For these reasons, PS was not included in SIPS. We do, however, note that 53% (*n* = 43) of patients with PS2 were SIPS2, reflecting the impact of systemic inflammation on functional status. We would advocate the use of SIPS alongside PS as part of the holistic assessment of patients and their management options.

A limitation of the current study is the lack of an external validation cohort. Unlike previous studies, however, we have carried out multivariate analyses of a range of prognostic scores, including their key individual components, in a single, well-defined patient cohort, from which we have identified a novel prognostic score framed in a clinical context. We strongly advocate further work incorporating independent validation of SIPS in this and other cohorts of patients being treated with ICIs. In particular, we recognise that a first-line, single-agent anti-PD-1 ICI is appropriate in other indications, including metastatic melanoma or renal cell carcinoma.^60^^,^^61^ First-line treatment options for metastatic NSCLC now also include ICIs in combination with cytotoxic chemotherapy regardless of PD-L1 status.^4^^,^^5^

Another concern commonly raised regarding these systemic biomarkers of inflammation is the effect of confounders. For example, in this patient group the use of steroids for the management of brain metastases or the presence of infection may raise NC and be the cause of early mortality themselves. Guidelines preclude the use of high-dose steroids when starting first-line ICI, however, meaning patients must be receiving <10 mg/day of prednisolone equivalent.^2^^,^^3^^,^^62^ Active infection is also considered a contraindication for the initiation of therapy. In this study, biomarkers were measured immediately pretreatment. In our institution it is recommended that patients have blood tests within 3 days before any treatment as part to of their routine clinical review. This gives confidence that the effect of these confounders is low. Further, amongst 169 assessable patients, there was no difference in the median NC between patients with known brain metastases treated with steroids in the preceding 2 weeks [(*n* = 8 (5%)] and all other patients [6.85 × 10^9^/l versus 6.30 × 10^9^/l, respectively (*P* = 0.192)]*.*

### Conclusions

The results of the present study demonstrate that biomarkers of systemic inflammation are reliable prognostic factors in patients with NSCLC expressing PD-L1 ≥50% treated with first-line pembrolizumab monotherapy. These findings support those made in other studies examining the prognostic utility of these measures in NSCLC and other cancers. The novel score, SIPS, stratifies survival in a clinically meaningful timeframe. Used alongside clinical assessment, this score may provide objective prognostic information and allow open discussion regarding the potential benefits of treatment in this patient group.
